# Hypergraph-based contrastive embedding and attention fusion for detection of skin cancer

**DOI:** 10.1038/s41598-026-43351-9

**Published:** 2026-03-09

**Authors:** Tathagat Banerjee, Prachi Chhabra, Manoj Kumar, Abhay Kumar, Kumar Abhishek, B. M. Ahamed Shafeeq

**Affiliations:** 1 Department of Computer Engineering, Indian Institute of Technology Patna, Bihar, India; 2 Department of Information Technology, JSS Academy of Technical education Noida, Noida, India; 3 School of Computer Science Engineering and Technology, Bennett University, Greater Noida, India; 4 Department of Computer Science and Engineering, NIT Patna, Patna, Bihar 800005 India; 5 Manipal Institute of Technology, Manipal Academy of Higher Education, Manipal, India

**Keywords:** Cancer, Computational biology and bioinformatics, Mathematics and computing

## Abstract

Skin diseases involve a spectrum of problems including infections, and malignancies. Melanoma, the deadliest kind of skin cancer, starts in melanocytes, which make melanin. Early detection is really important, but it’s hard since the visual indications are often quite little and there is a big class imbalance in diagnostic datasets. The proposed C2G-HFMTA framework consists of three hierarchical levels: (a) an overall contrastive learning (CL) framework, (b)two major feature learning branches, namely the Graph Contrastive Embedding Framework (GCEF) and the High-dimensional Feature with Multimodal Transformer Attention (HFMTA), and (c) attention and fusion sub-modules including Hypergraph Bi-Convolutional Attention and Multiscale Transformer Attention, which operate within these branches to enhance discriminative representation learning. The proposed method demonstrates strong performance on benchmark dermoscopic datasets and has the potential to support computer-aided diagnosis systems, subject to further may support future computer-aided diagnosis systems validation and real-world testing. We have used Clustered Class-Based Segmentation (CCBS) for changing the training distributions. Our Class-Based Contrastive Loss (CBCL) works directly on original dermoscopic pictures, that preserves the semantic integrity of the images while making it easier to tell the difference between classes. Our framework outperforms several recent CNN- and transformer-based baselines in controlled experimental settings. It gets 93.2% accuracy and a 92.9% F1-score, and it does well on minority classes. Experiments were conducted on the HAM10000 dataset containing 10,015 dermoscopic images across seven diagnostic categories, using a stratified train–validation–test split of 70%–10%–20%. Performance was evaluated using accuracy, precision, recall, and F1-score, using five-fold stratified cross-validation to ensure robust performance estimation. Ablation experiments show that grouping, cross-branch fusion, and semantic-guided attention are important.

## Introduction

Early detection of skin cancer is essential for improving patient survival and reducing treatment complexity, particularly among populations with lighter skin tones^1^. Melanoma, although less frequent than other skin cancers, is the most aggressive and life-threatening form^2^. Skin cancer, which encompasses multiple types of lesions such as basal cell carcinoma, squamous cell carcinoma, and melanoma, continues to present diagnostic difficulties owing to high inter-class similarity and intra-class variability in lesion appearance^3^. As a result, reliable and objective computer-aided diagnosis (CAD) systems have become increasingly important in supporting clinical decision-making^4,5^.

Recent advances in deep learning have significantly improved automated skin lesion analysis using dermoscopic images. Convolutional neural networks and transformer-based architectures have demonstrated strong classification performance; however, their effectiveness is often limited by inherent characteristics of dermoscopic datasets. Automatic skin cancer screening devices are in high demand due to their accuracy. These devices often collect two types of photos: dermatoscopic images from specialty equipment in pathology centres, which require dermatologist assessment, and digital photographs taken at home using regular cameras. Skin cancer screenings can be performed in the comfort of one’s own home using software automated screening support systems that assist lesion-level classification without explicit ROI annotation^6^. Several recent focused studies assess and extend augmentation techniques specifically for dermoscopy images^7^. provides a comparative analysis of dermoscopy augmentation methods and introduces a wavelet-packet augmentation that preserves lesion textures while diversifying training samples, illustrating that transform-based augmenters can be effective alternatives to blind geometric/photometric augmentations. Building on augmentation with learned generative models^8^, proposed a GAN-based dermoscopy augmenter that uses a hybrid content/SSIM/L1 loss to improve image fidelity and to reduce artifacts common in GAN outputs. Both studies confirm that augmentation—whether model-based or transform-based—can improve classifier robustness, but they also underscore practical limitations such as increased training complexity, potential semantic drift in generated examples, and sensitivity to loss-function choices and model stability, especially when may support future computer-aided diagnosis systems subtle features must be preserved. While recent studies have explored augmentation strategies and representation learning techniques to mitigate class imbalance, these approaches are often applied independently and may not fully exploit relationships across lesion categories. Moreover, generative augmentation and heavy transformation pipelines may introduce semantic drift or increase training complexity. These observations motivate the development of integrated representation-level frameworks that can leverage class relationships while preserving semantic fidelity of dermoscopic images. In this context, we propose C2G-HFMTA, a Contrastive Learning (CL) based architecture that combines class-aware data structuring, graph-supervised embedding, and multimodal attention to improve minority-class discrimination. Although prior works have explored class rebalancing, graph-based learning, contrastive objectives, and attention mechanisms independently, there is limited work that integrates these strategies within a unified contrastive framework for highly imbalanced dermoscopic datasets. C2G-HFMTA provides a potential foundation for exploring similar integration strategies in other imbalanced biomedical imaging domains.

The framework comprises three major contributions as described below:

- Presentatio of a Clustered Class-Based Segmentation (CCBS) Strategy: We introduce a new method of restructuring the training data which divides it into clusters of training data related to specific classes. This method is systematic in the control of the representation of the dominant, middle, and minority classes and is a direct response to the problem of class imbalance and guarantees the creation of even-handed learning within the whole range of labels.
- Creation of the Graph Contrastive Embedding Framework (GCEF): In this paper, a specialized framework based on a HyperGraph with Bi-partite Convoluted Attention (HyperGraph-BiConvAtt) will be presented. This process allows producing highly discriminative, class-conscious embeddings, which captures more complex higher-order relations. It is important to note that this can be done without the computational cost of data augmentation or even the unpredictability of adversarial training.
- Multimodal Feature Fusion Design through HFMTA: We submit the high-dimensional Multimodal Features fusion architecture, which is a state-of-the-art feature-fusion module, the High-dimensional Feature with Multimodal T-block Attention (HFMTA). This module increases the capacity to identify fine-grained, subtle trends in minority classes that would otherwise remain unnoticed by the conventional feature extraction methods.

Unlike traditional contrastive learning methods that depend on augmented image pairs, the proposed Class-Based Contrastive Loss (CBCL) operates directly on original dermoscopic images and defines positive and negative relationships based on lesion categories within a supervised graph structure. This design preserves semantic fidelity while promoting compact intra-class representations and well-separated inter-class boundaries. Furthermore, the bipartite attention mechanism in the hypergraph framework enables asymmetric information propagation, reducing feature over smoothing and improving representation learning for minority classes.

The proposed C2G-HFMTA framework is evaluated on the HAM10000 dataset using five-fold cross-validation. Experimental results demonstrate consistent improvements over multiple convolutional and transformer-based baselines, particularly in macro-level performance metrics and minority-class recognition. Qualitative analyses using Grad-CAM and t-SNE visualizations further illustrate enhanced feature localization and class separability. Overall, this work presents a unified, imbalance-aware learning framework that improves dermoscopic image classification. particularly in macro-level performance metrics and minority-class recognition. Qualitative analyses using Grad-CAM and t-SNE visualizations further illustrate enhanced feature localization and class separability. Overall, this work presents a unified, imbalance-aware learning framework that improves dermoscopic image classification.

The rest paper is structured as Sect.  2 presents recent reviews of related studies while Sect.  3 describes the proposed architecture. Section  4 discusses experiment specifications, dataset characteristics, results, and ablation study followed by Sect.  5, which represents the conclusion.

## Literature review

A shift toward representation-level solutions has brought contrastive learning to the forefront of medical image analysis. Methods such as SimCLR^22^, SupCon^23^, Barlow Twins^24^, and MoCo v3^25^ learn invariant feature representations by pulling together embeddings of augmented views of the same sample while pushing apart dissimilar instances. Although effective for natural images, these approaches depend heavily on strong data augmentations to define positive pairs, which can distort subtle color, texture, and boundary cues that are diagnostically critical in dermoscopic imaging. Furthermore, these methods do not explicitly model class-wise relationships, limiting their ability to enforce compact intra-class clustering and sufficient inter-class margins under severe class imbalance.To mitigate these limitations, graph-based contrastive learning frameworks such as GraphCL^26^ incorporate relational information between samples to preserve contextual structure, which improves robustness under skewed label distributions. However, pairwise graph constructions restrict the modeling of higher-order correlations among multiple minority-class samples. Hypergraph-based methods, including HGNNs^27^, HyperGCL^28^, and SHGNet^29^, extend graph learning by capturing group-wise relationships through hyperedges, enabling better representation of complex semantic neighborhoods. Despite this advantage, most existing hypergraph contrastive frameworks remain self-supervised or weakly supervised and do not directly integrate class-aware contrastive objectives, which are essential for maximizing minority-class separability in clinical datasets. Recent imbalance-aware contrastive methods^30–33^ attempt to address this gap through reweighting or modified sampling strategies, yet they typically operate without explicit relational modeling of sample clusters and may fail to preserve fine-grained inter-class boundaries. Focal Loss and Label-Distribution-Aware Margin (LDAM) loss are measures to mitigate loss-level imbalances in loss-sensitive deep learning optimization by reweighting gradients or adjusting the decision-margin. Although these methods are effective in the context of enhancing the ability of minority-class recollection, the methods assume fixed feature representations and these techniques do not explicitly impose relational structure or intra-class compactness on the embedding space. Conversely, C2G-HFMTA focuses on the imbalance in the representation-learning level to jointly learn the class-aware contrastive goals and hypergraph-based relational supervision, so that the minority-class separability can be enhanced before final classification. In addition, recent class-aware contrastive learning methods introduce label-conditioned positive pair selection, adaptive margins, or prototype-based supervision to improve intra-class compactness and inter-class separation under imbalance. While these strategies enhance discrimination at the instance or class-prototype level, they still rely on pairwise relationships and do not model higher-order sample groupings or structural dependencies among minority-class instances. Moreover, most class-aware contrastive approaches treat class supervision as a weighting or sampling mechanism rather than embedding it into the relational structure of representation learning itself. Consequently, they may struggle to preserve local semantic consistency within sparse minority classes when visual variations are high, as commonly observed in dermoscopic datasets. The limitations motivate the proposed supervised hypergraph-based contrastive embedding in C2G-HFMTA, where class-clustered segmentation and class-based contrastive loss are jointly optimized to explicitly enhance minority-class compactness and inter-class discrimination.

Although prior graph- and hypergraph-based contrastive frameworks model relational structures among samples, they typically construct similarity graphs from feature proximity or data augmentations and optimize self-supervised objectives without explicit class-aware constraints. In contrast, the proposed GCEF in C2G-HFMTA operates on class-clustered sample groups and employs supervised class-based contrastive loss, where positive and negative relations are defined by lesion categories rather than augmentation-induced similarity. Moreover, the use of bipartite hypergraph attention between anchor and contrast nodes enables asymmetric information propagation, which differs from conventional uniform message passing in existing hypergraph neural networks and is specifically designed to enhance minority-class representation.

In parallel, multimodal and attention-based fusion strategies have been increasingly explored to enhance skin lesion classification by integrating complementary visual and semantic cues. Transformer-based fusion networks and cross-attention mechanisms have shown improved modality alignment and interpretability by dynamically weighting discriminative regions and feature channels^34–37^. Recent dermatology-specific models further incorporate hierarchical attention and multi-scale feature aggregation to improve lesion boundary sensitivity and diagnostic transparency^38–40^. While these approaches improve overall classification accuracy and visual interpretability, they generally assume sufficiently balanced training distributions and optimize global decision boundaries, without explicitly addressing representation degradation for under-represented lesion categories. Consequently, attention weights may still be dominated by majority-class patterns, limiting sensitivity to rare but clinically critical cases. Moreover, most fusion frameworks treat representation learning and modality alignment as separate stages, which can weaken cross-modal consistency under severe imbalance. These observations further motivate the proposed High-dimensional Feature with Multimodal T-block Attention (HFMTA) module, which directly conditions visual feature fusion on class-aware graph embeddings, enabling semantic-guided attention that prioritizes minority-class discriminative patterns during feature integration.

Ranking Diffusion Transformer (RDT)^41^, a Ranking Visual Encoder is suggested that consists of forward and inverse ranking attention and a ranking loss combined into a diffusion framework to reinforce discriminative visual representation and vision-language alignment to fine-grained image captioning, Whereas the method has been shown to have better descriptive performance and cross-domain generalization. It presented an extra computational cost of multi-head ranking attention and diffusion-based optimization. In the face alignment application, the Multi-Order High-Precision Hourglass Network^42^(MHHN) takes advantage of the higher-order cross-layer geometry correlations and Heatmap Subpixel Regression to harness the higher-order localization performance and higher pose variations; however, at the cost of model complexity. Equally, the Multiorder Multiconstraint Deep Network (MMDN)^43^ added an Implicit Multorder Correlating Geometry-aware module, and Explicit Probability-based Boundary-Adaptive Regression strategy to spatialchannel correlations and impose global shape constraints. Although this framework indicates reduced landmark localization error on difficult datasets. The series of works show together that multiorder correlations and structured constraints can be combined to a significant improvement in performance, but with increased computational cost.

Recent dermatology-specific architectures such as PABT-Net^44^ and BCB–CSPA^45^ enhance multi-scale feature extraction and attention-based fusion; however, these models primarily focus on architectural refinement and do not explicitly incorporate class-aware contrastive objectives or relational supervision for imbalance mitigation. These challenges are particularly evident in datasets such as HAM10000, where class skew and limited minority samples hinder reliable representation learning and fair performance evaluation.

In contrast, C2G-HFMTA addresses the above-mentioned gaps by integrating:

- CCBS to create balanced learning clusters.
- GCEF with supervised class-based contrastive loss (CBCL).
- A HyperGraph-BiConvAtt module that uses bipartite attention over anchor and contrast nodes.
- A multimodal fusion block (HFMTA) that aligns semantic embeddings with visual DenseNet features using attention.

This integrated design reduces dependence on synthetic augmentation and enables class-discriminative embedding through relational supervision and modality-aligned attention, which is particularly beneficial under extreme class imbalance. While class clustering and attention-based fusion have been explored independently in prior studies, their joint integration with supervised hypergraph contrastive embedding and semantic-guided multimodal attention within a unified end-to-end framework constitutes the novelty of this work.

## Methodology

**Fig. 1:**
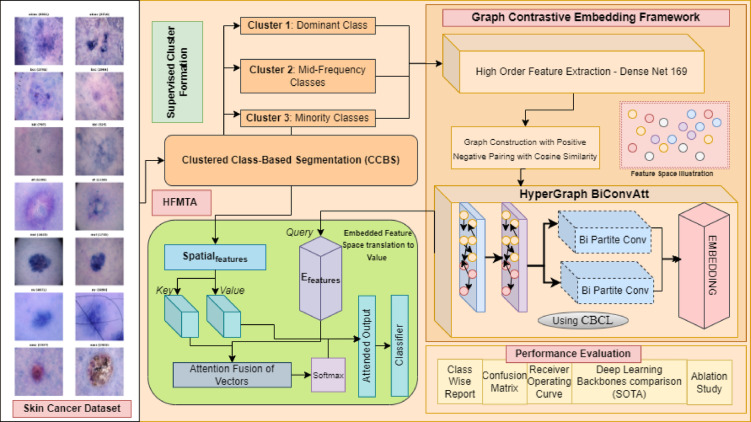
Class-clustered contrastive embedding and high-dimensional feature attention residual network.

We suggested C2G-HFMTA framework to overcome the widespread issue of extreme imbalance of classes and subtle visual differences at inter-class in skin lesion classification. This is a multi-stage systematic method that tries to eliminate learning biases against dominant classes, which in turn results in poor generalization and high error rates of underrepresented minority classes like Vascular Lesions and Basal Cell Carcinoma. These limitations are mitigated by the architecture that incorporates three hierarchical levels: the Clustered Class-Based Segmentation (CCBS) to restructure data, the Graph Contrastive Embedding Framework (GCEF) to learn relational features, and the High-dimensional Feature with Multimodal Transformer Attention (HFMTA) to learn semantic features in a guided manner. The combination of these components into an integrated end-to-end framework is shown in Fig. 1. The aim of the proposed methodology is to enhance discriminative representation learning and increase diagnostic accuracy in all 7 categories of the HAM10000 dataset.

The flow of the methodology is as follows:

### Data preprocessing and restructuring

The process begins with the Clustered Class-Based Segmentation (CCBS**)**. CCBS divides the dataset into segments based on the distribution of classes to ensure that its representation is balanced during training. It directs a common training process to dynamically distribute attention to clusters according to their class frequencies. This stage restructures the training dataset (HAM10000) into three specific groups based on sample frequency:

Cluster 1 (Dominant Class): Specifically manages the most frequent class (Melanoma) to prevent model overfitting and over-representation.

Cluster 2 (Mid-Frequency Classes): This cluster contains medium-sized classes with 1000 and 1500 images in each class. Nevus, Actinic Keratosis, and Basal Cell Carcinoma belong to this cluster.

Cluster 3 (Minority Classes): Focuses on under-represented classes such as Vascular Lesions and Dermatofibroma, ensuring they receive distinct training attention. These under-represented classes face difficulties extracting features and demonstrating weak classification performance because they are not properly featured in the learning process.

Its fundamental purpose consists of reducing the distances between samples belonging to the same class and increasing the distance between samples of different classes. The process produces densely clustered data points, which allow images from the same category to remain near one another but separate these images from different classes.

### Feature extraction

The DenseNet201 model is applied as the backbone of high-order feature extraction since it is more efficient than other models in extracting important morphological features at different levels of spatial details. The model operates on input dermoscopic images (usually 224 × 224 pixels) in C2G-HFMTA framework by process consists of a deep layered architecture where each layer is fed directly by all the previous layers. This dense connectivity guarantees the highest information flow and the vanishing-gradient problem is alleviated, since the network is able to capture fine-grained visual features required to detect fine lesion characteristics. In particular, the feature vector of 1664 dimensions obtained on the last layers of DenseNet201 can be used as the baseline visual representation. The extracted features are then used in Helmholtz dual directions: they form the starting graph nodes to the Graph Contrastive Embedding Framework (GCEF) and are the Key and Value inputs of the HFMTA multimodal attention block so that they could be used to implement semantic-guided fusion.

### Graph contrastive embedding framework (GCEF)

The extracted features are passed into the GCEF, which employs Contrastive Learning to organize the embedding space. It brings similar images nearer to one another in their feature space while consistently separating images belonging to different classes. Its fundamental purpose consists of reducing the distances between samples belonging to the same class and increasing the distance between samples of different classes. The process produces densely clustered data points, which allow images from the same category to remain near one another but separate these images from different classes.

**Fig. 2:**
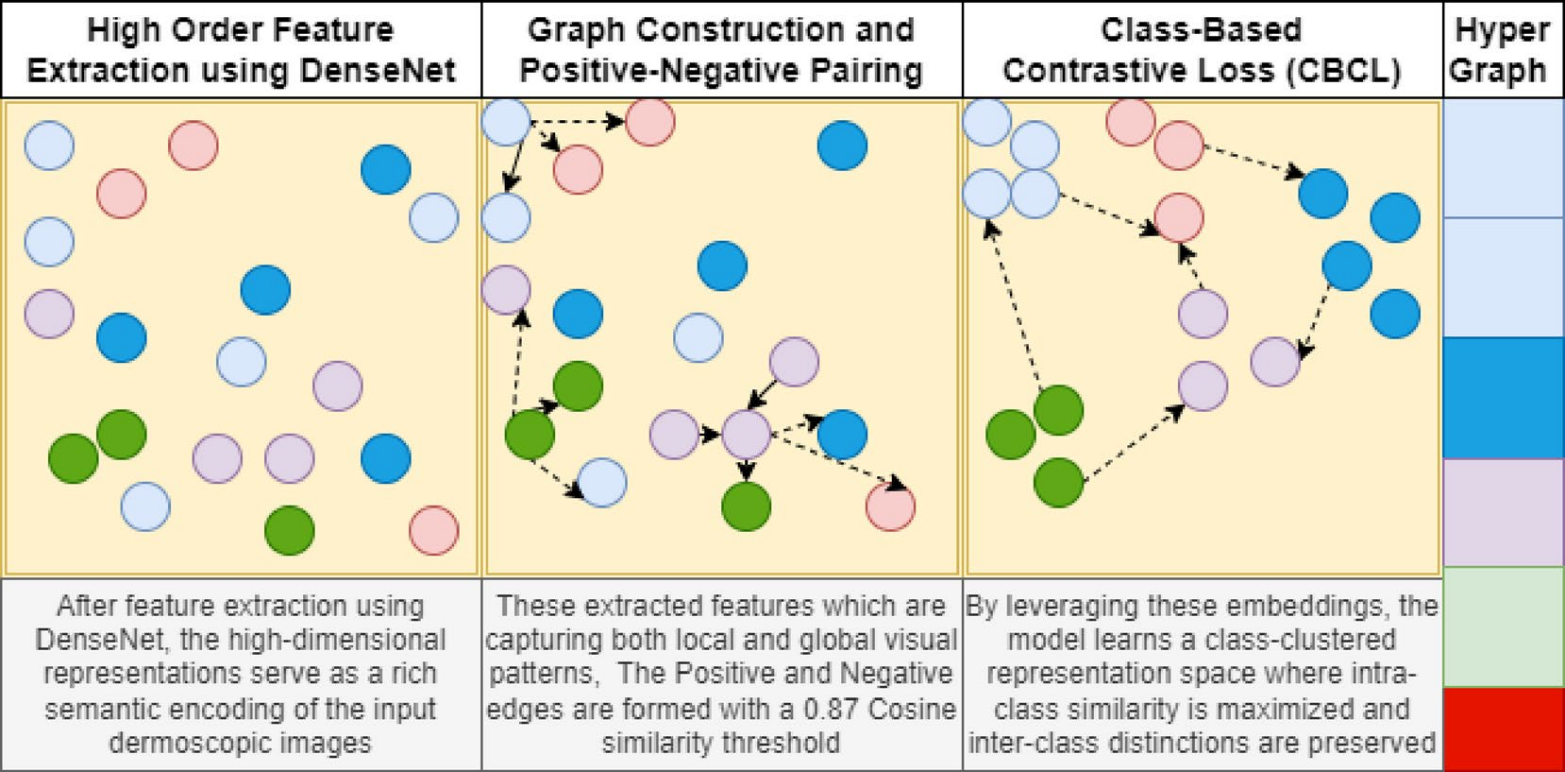
Class-wise graph contrastive embedding framework.

Figure 2 displays, each step of the feature embedding with contrastive edges of positive and negative similarity and finally the Hyper Graph provides the segregated embedding space for downstream classification. The strategy benefits class-imbalanced situations by stopping the model from developing class preferences, thus allowing it to discover generalizable features across all classes. When visual elements are placed in this optimized dimensional domain that enhances class separation.

- Visual features are extracted as the initial process in the GCEF pipeline through DenseNet201 using Algorithm 1. DenseNet201 demonstrates excellence in architectural design, implementing dense connections to share information across all previous layers. Retention in the feature maps enables the system to retain critical details at different levels, which is essential for analyzing complex skin lesion images. The dense features serve to construct the graph structure before triggering contrastive learning.

**Algorithm 1:**
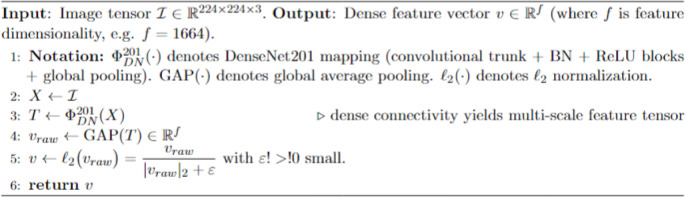
High-order visual feature extraction via DenceNet201.

- Graph Construction and Positive-Negative Pairing: After extraction, we build a graph network where each image becomes a node, and the edge strength reveals similarity between them. The graph-building process utilizes cosine similarity to calculate image pairwise similarity through their vector angle comparison. The images fulfill the grouping requirement for the positive relationship when their cosine similarity measure reaches 0.87 or above. Images that show less than this specified cosine similarity threshold obtain a negative relation status, thus receiving different class assignments.
  The feature space development of the model relies heavily on positive and negative pairing in contrastive learning shown by Algorithm 2. The model learns from positive pairs containing images within the same class, while negative pairs are images between different classes that need separation in embeddings.

**Algorithm 2:**
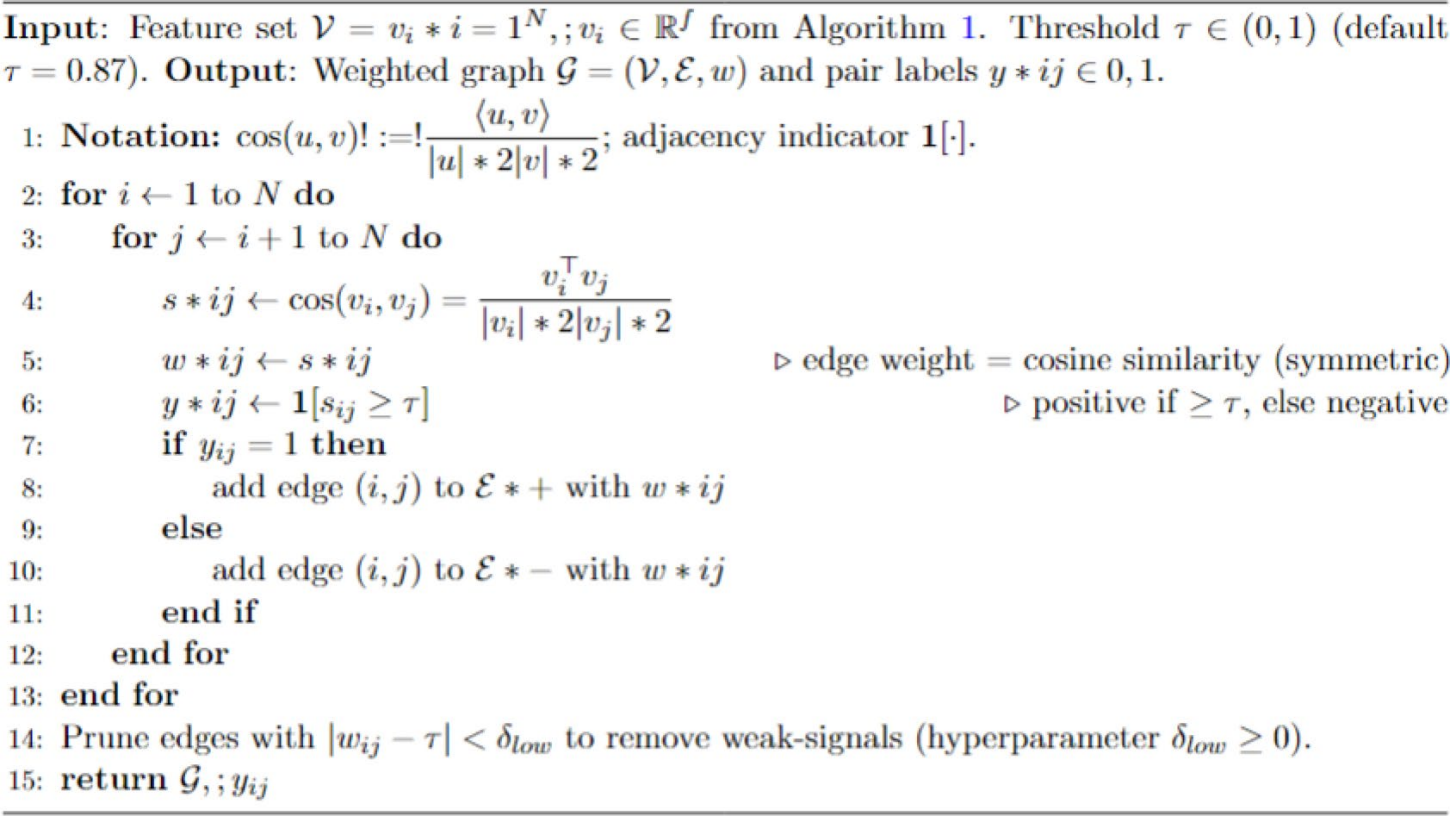
Graph construction positive/negative pairing.

- Class-Based Contrastive Loss (CBCL): A class-based contrastive loss technique focuses on bringing pictures of the same category closer and forcing unrelated images from different groups further away from each other. It defines positive and negative relationships based on lesion categories to promote compact intra-class clusters and well-separated inter-class boundaries. The contrastive loss maintains balanced learning representations when dealing with class imbalance situations. This loss is evaluated by equation (i).
  i $$L=\frac{1}{2N}{\sum}_{i=1}^{N}\left[{y}_{i}\cdot\left(|{z}_{i}-{z}_{i}^{+}{|}^{2}\right)+\left(1-{y}_{i}\right)\cdot{\mathrm{max}\left(0,m-|{z}_{i}-{z}_{i}^{-}|\right)}^{2}\right]$$

N is the number of image pairs.

${y}_{i}$Is the label indicating whether the pair is positive (1) or negative (0).

${z}_{i}$ is the feature vector of the anchor image.

${z}_{i}^{+}$ is the feature vector of the positive image (same class).

${z}_{i}^{-}$ is the feature vector of the negative image (different class).

$m$is the margin that defines the minimum distance between negative pairs.

This method directly improves embeddings to bring the same class together and push samples of other classes away, all without changing the content of the images as illustrated in algorithm 3.

**Algorithm 3:**
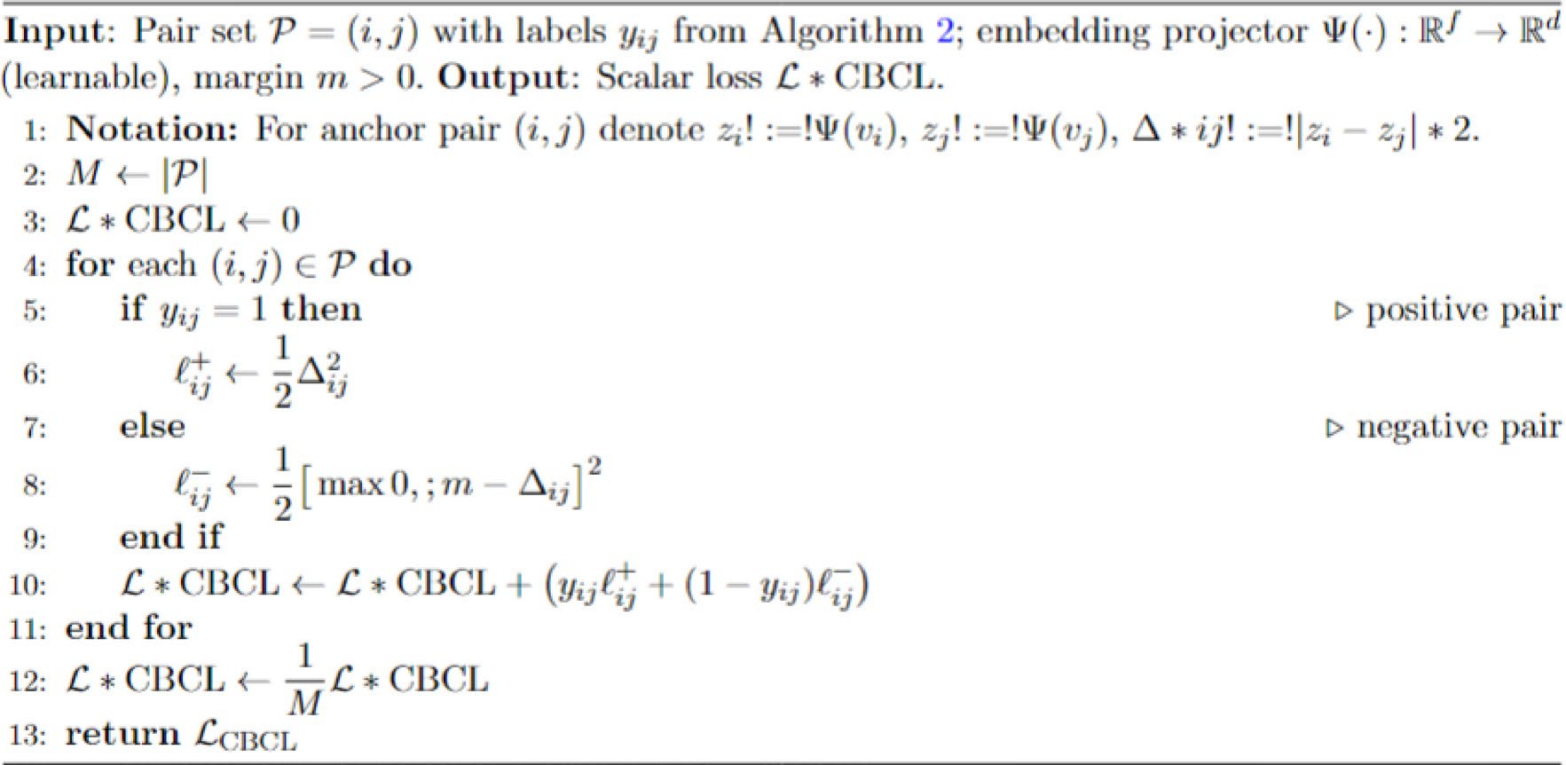
Class-based contrastive loss (CBCL)—Pairwise formulation

- Hypergraph Bi-partite Attention: The HyperGraph-BiConvAtt mechanism operates as a specialized message-passing layer within the Graph Contrastive Embedding Framework (GCEF) to optimize feature representations under extreme class imbalance. As detailed in Algorithm 4, this process begins by partitioning the graph into two distinct sets: anchor nodes, which represent the current mini batch of images being trained, and contrast nodes, which consist of pre-clustered samples from the broader dataset.

**Algorithm 4:**
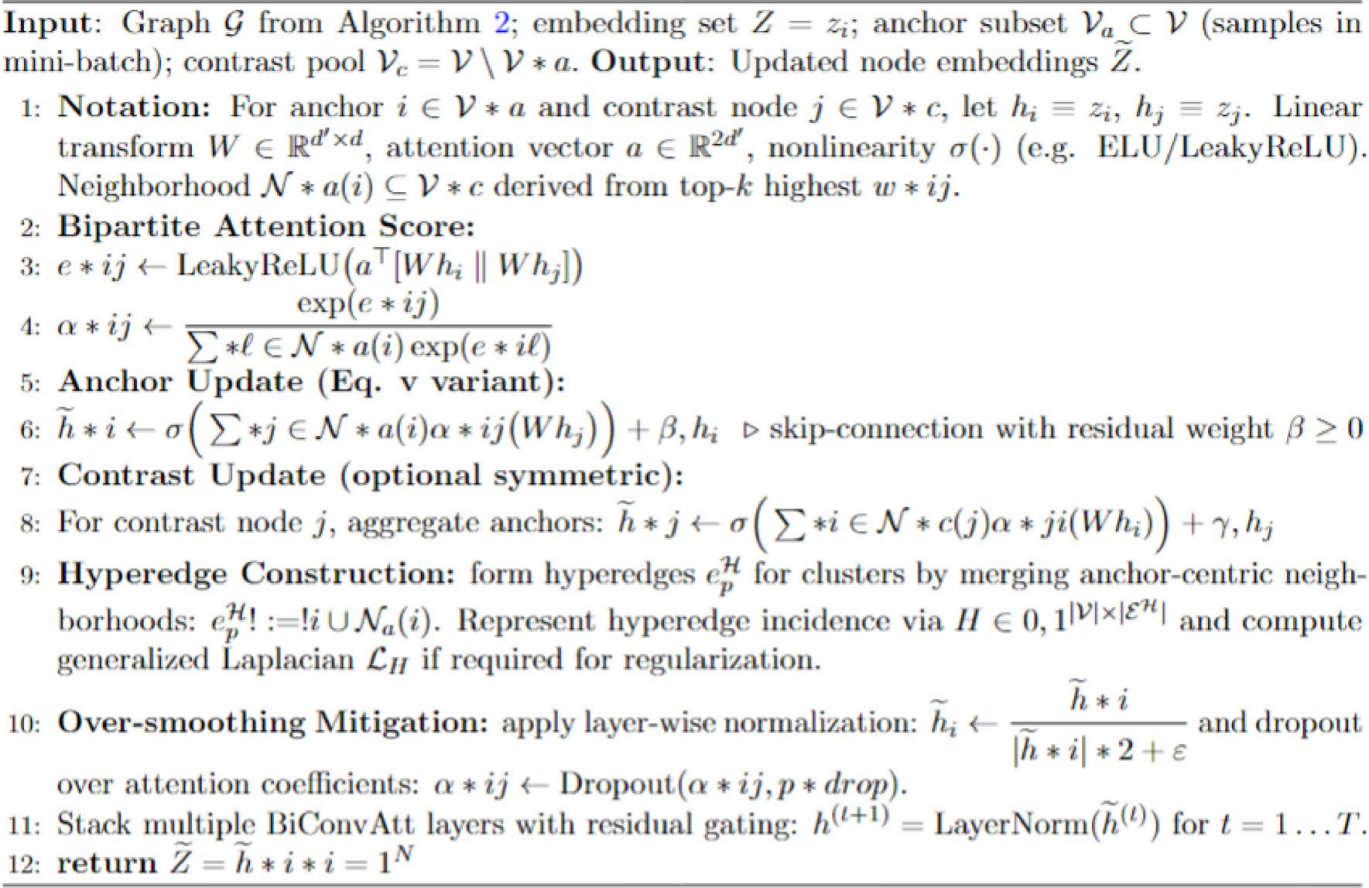
HyperGraph-BiConvAtt: Bipartite hypergraph convolution with attention.

The mechanism employs a Graph Attention Convolution (GATConv) to facilitate asymmetric information propagation, where attention coefficients $\left({\alpha}_{ij}\right)$are calculated between these two partitions to prioritize the most semantically relevant neighboring information. By focusing on these structured bipartite interactions rather than uniform global message passing, the framework prevents “over-smoothing” of features, a common issue where distinct class signatures are lost and specifically reinforces the unique morphological signatures of minority classes. This targeted propagation ensures that the learned embeddings for rare lesions remain discriminative and well-separated from dominant categories, enabling more accurate downstream classification.

### High-dimensional feature with multimodal transformer attention (HFMTA)

The High-dimensional Feature with Multimodal Transformer Attention (HFMTA) module serves as the downstream classification engine of the framework, designed to unify deep visual features with high-dimensional semantic embeddings from the graph contrastive learning phase. As outlined in Algorithm 5, this module utilizes the semantic embeddings derived from the Graph Contrastive Embedding Framework (GCEF) as query vectors to guide the interpretation of visual features initially extracted from the DenseNet201 backbone. A critical component of this process is the Residual Enhancement block, which incorporates a two-layer feedforward network with skip connections to preserve the semantic clarity of the embeddings and prevent “over-smoothing” in high-dimensional space. This architecture ensures that the model maintains delicate morphological differences between similar lesion classes, which is essential for accurate multi-class classification.

**Algorithm 5:**
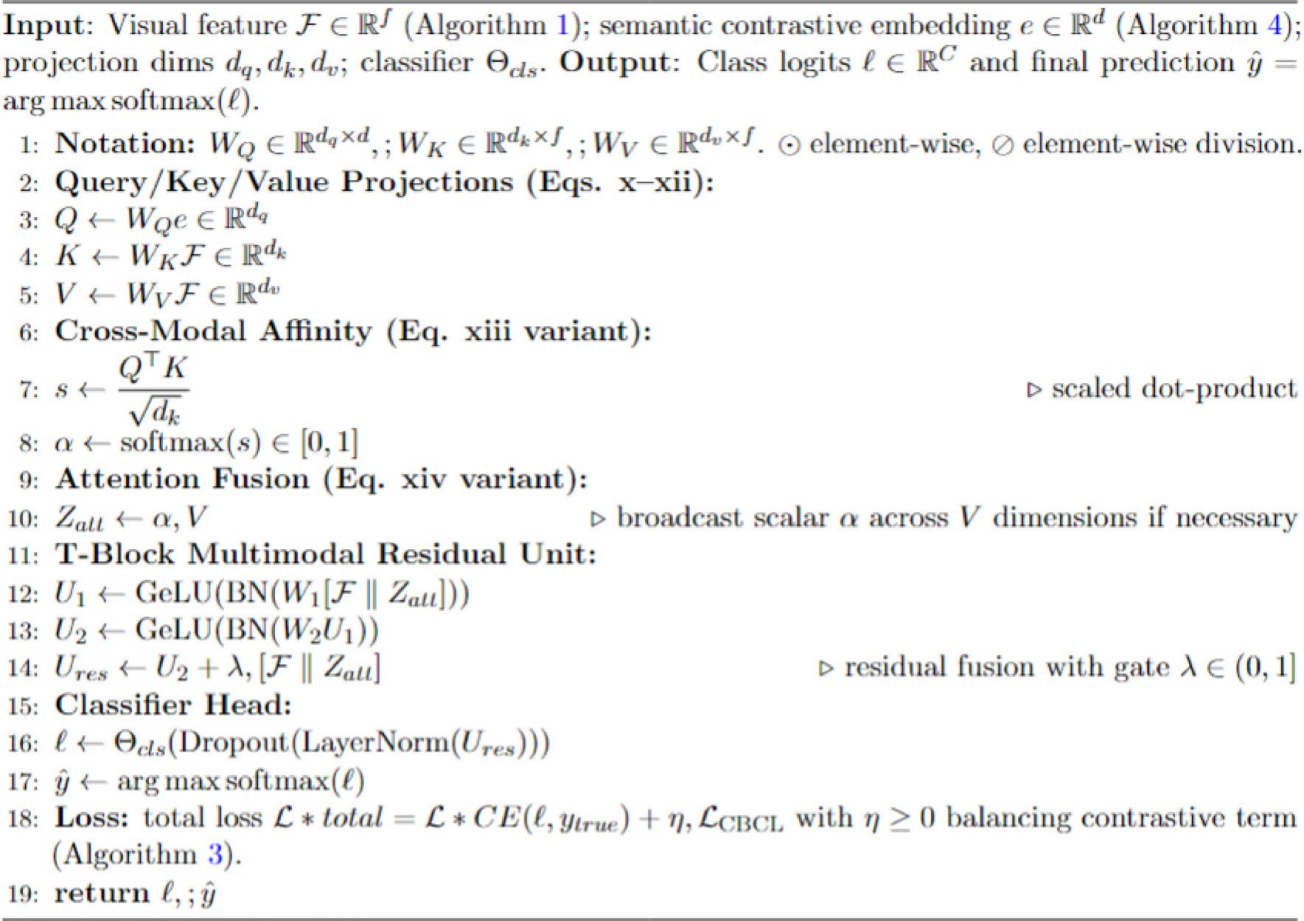
HFMTA: High-dimensional feature fusion with multimodal T-block attention.

**Fig. 3:**
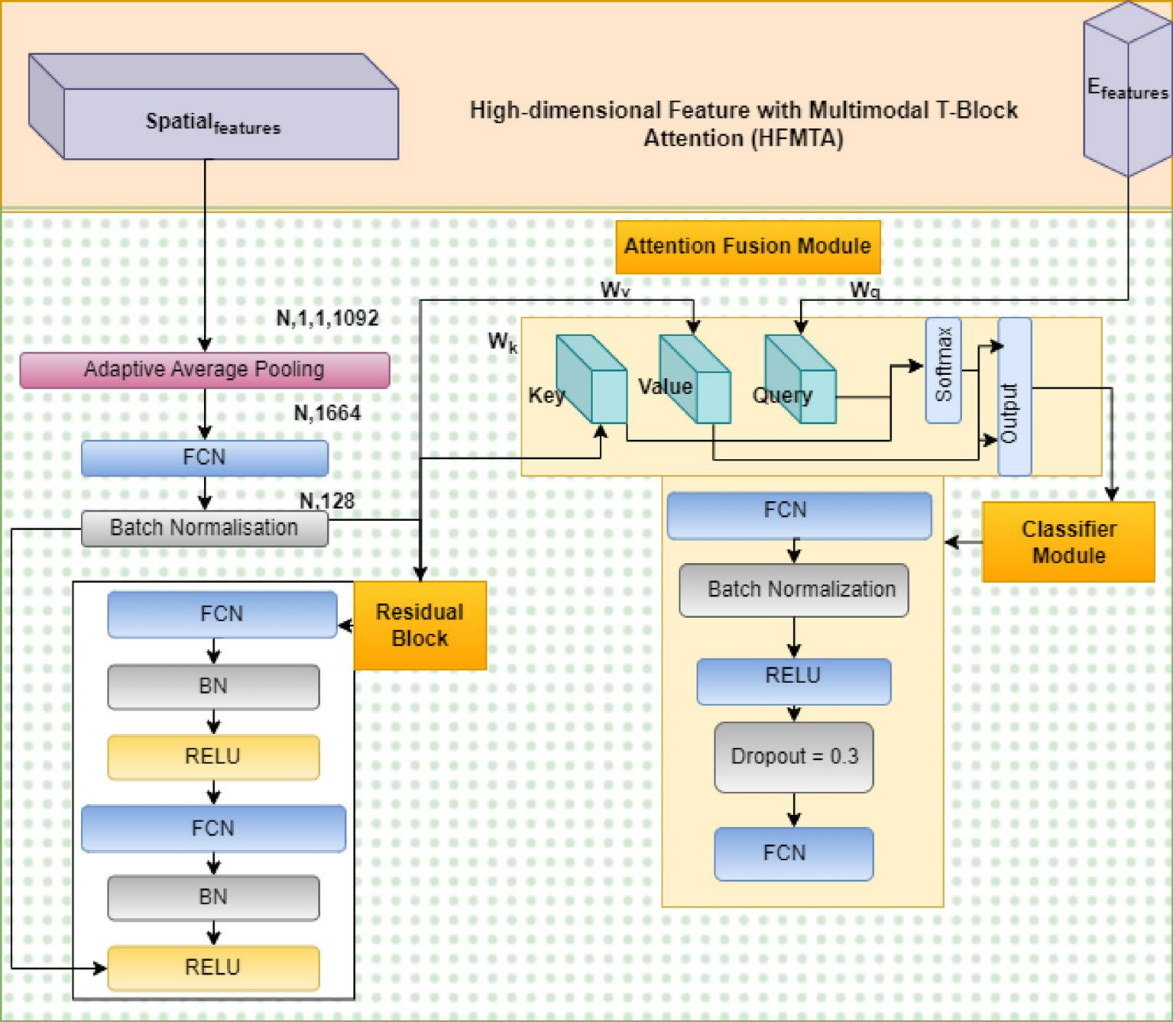
High-dimensional feature with multimodal T-block attention (HFMTA) framework.

The visual representation of this process in Fig. 3 illustrates an asymmetric fusion scheme where the semantic class distribution influences the generation of “soft attention” weights. Specifically, the semantic embeddings are projected into a Query vector ($W_Q$), while the 1664-dimensional visual features from DenseNet are transformed into Key ($W_K$) and Value ($W_V$) vectors. By performing element-wise interactions between these vectors, the HFMTA module selectively filters significant spatial and semantic dimensions, effectively highlighting small morphological clues required to diagnose rare diseases. The resulting fused output is then passed through a final classification head—comprising linear transformations, dropout, and normalization—to project the high-dimensional representation into the final seven-class diagnostic space of the HAM10000 dataset.

### Classification

The fused representation is passed through a final classification head consisting of linear transformations and activation layers to project the data into the final class label space. The entire framework is optimized end-to-end under a unified training objective.

## Results and discussion

This section describes the real-life implementation of the proposed framework of class imbalance awareness and provides an extensive discussion of the demonstrated performance of the proposed framework on the HAM10000 dataset. We utilized PyTorch version 2.1.0 on a Linux system that includes an NVIDIA RTX 3090 GPU (24GB VRAM), 64GB DDR4 RAM, AMD Ryzen 9 5950 × 16-core CPU, and CUDA 11.7 for processing. AMP speeds up training sessions and reduces GPU memory requirements during training. The process of data preparation, such as preprocessing, data partitioning plan, and cross-validation plan are also briefly outlined. After this, the quantitative and qualitative findings of the experiments are discussed systematically in comparison to the proposed approach and the conventional CNN and transformer-based models in terms of the standard evaluation measures of accuracy, precision, recall, F1-score, and performance per class. The particular attention is paid to the possibility of the model addressing the issue of the imbalance of classes and enhancing the recognition of minority classes. The discussion also explains the input of main architectural elements by the ablation analysis.

### Data description

HAM10000 dataset known as Human Against Machine with 10,000 training image, is one of the well-known and highly applicable benchmark datasets in the dermatological image analysis and skin lesion classification domain. It includes 10,000 dermoscopic images in seven diagnostic relevant categories including melanoma (mel), melanocytic nevi (nv), basal cell carcinoma (bcc), actinic keratoses and intraepithelial carcinoma (akiec), benign keratosis-like lesions (bkl), dermatofibroma (df), and vascular lesions (vasc). The presence of equal representation of the types of malignant and benign lesions permits the strong testing of classification algorithms. The dataset^17^ combines the samples of two large clinical centers in Austria and Australia, providing a wide range of imaging conditions, skin types, and the location of the lesion, which positively affects the extrapolability of machine learning models. These are supported by expert-verified ground truth annotations, which are acquired in histopathology, expert consensus, or clinical follow-up. It guarantees a high degree of diagnostic reliability. Other metadata indicators like patient age, sex and the anatomy location are also available, and multimodal models can be constructed, which extend beyond the classification of pure images. HAM10000 dataset is crucial in aiding the creation of leading AI models and benchmarking advanced AI models of parts of lesion classification, segmentation, and explainable AI, among others, contrastive learning, and embedding visualizations. It is a resource that cannot be ignored by researchers who seek to develop credible and understandable AI systems in dermatology because of its clinical realism, extensive labeling, and scale. The problem of overfitting is frequent, especially when the model is over-specialized on the training set, especially in cases of complex architecture. The disproportion may cause biased models, which are biased to the majority group, resulting in inaccurate measurements. Moreover, research studies reporting artificially high accuracies are common in state-of-the-art research studies which use the full dataset to report artificially high results, particularly in cases where the imbalance of classes is not adequately addressed, and inflated measures of performance are reported instead of true performance in the real world.

**Fig. 4:**
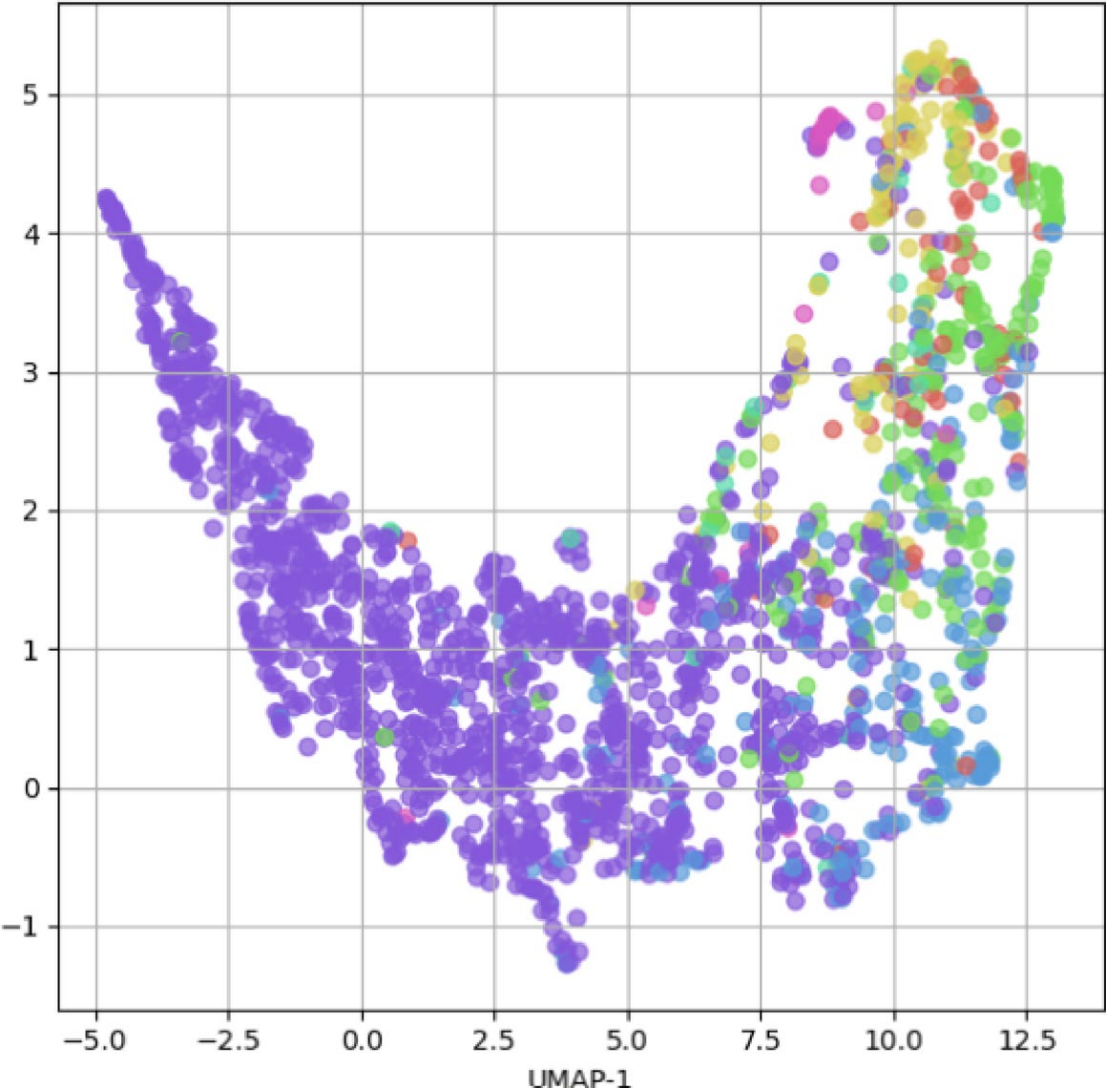
The all-class reorientation after 600 epochs HyperGraph-BiConvAtt.

The class structuring and embedding problems arise when the feature space doesn’t effectively separate different classes due to the lack of distinguishable kernels as shown by the Fig. 4. Even after 600 epochs, the model is unable to create distinctive boundary structure on the test data thus eventually would lead to errors in classification like overfitting and underfitting, which result from improper class encoding or the inability to capture nuanced differences in the feature space.

### Training procedure

The training process of C2G-HFMTA framework employed a systematic multi-stage approach. It resolves typical dermoscopic image classification issues such as imbalanced data, model overfitting and poor generalization performance. The standardized resolution preprocessing for all dermoscopic images reached 224 × 224 pixels and then proceed with normalization using per-channel mean subtraction and standard deviation normalization set to mean: [0.485, 0.456, 0.406], std: [0.229, 0.224, 0.225]. Extensive data augmentation methods using random rotation from − 25° to + 25° combined with width and height shifts of ± 10% as well as horizontal and vertical flips and brightness and contrast jittering (± 20%) and zoom scaling (range = 0.8–1.2) and cutout augmentation prevent overfitting in the limited and imbalanced data sets. A coping mechanism for the class imbalance issue, especially with underrepresented lesion types such as melanoma, employs Clustered Class-Based Segmentation through DBSCAN (ε = 0.5, min samples = 5) clustering to create sample similarity groups followed by stratified resampling across clusters to achieve fair learning contributions during training.

The Graph Contrastive Embedding Module (GCEM) created a similarity graph through which the nodes signify image embeddings connected by cosine similarity between same-class and cross-class pairs. During each batch the model generates positive and negative pairs through K-nearest neighbor sampling with K set to 5 and trains with NT-Xent contrastive loss that employs a temperature scaling of τ = 0.5 to achieve the desired separation in the latent space. The Hybrid Feature Map Temporal Attention (HFMTA) operates as the base component that utilizes a 5-stage convolutional encoder equipped with 3 × 3 kernels which performs sequential ReLU activation and batch normalization. It expands filter depths from 64 to 128 to 256 to 512 and concludes at 1024.

The model includes several attention heads in its temporal unit to select and combine features from both spatial positions and different semantic stages. The model uses layer normalization as its first step to ensure smooth training of attention blocks. A final 3-layer dense classifier with dropout (rate = 0.4) outputs SoftMax probabilities. We trained using Adam with beta1 set to 0.9, beta2 at 0.999, small value epsilon at 1x e^-8^ and 1x e^-5^ L2 weight decay. The starting value of 1x e^-4^ for the learning rate controls local minima to push for faster and more precise model convergence. The training continues for 20 phases with 32 examples per batch until the model stops making progress in validation F1-score detection for 15 epochs.

Each experiment split follows a five-fold validation approach for balanced training of all minority groups. Our performance evaluation includes per-class and macro-level scores across five folds to determine Accuracy, Precision, Recall, F1-Score, and AUC-ROC results. We provide Grad-CAM heatmaps and embedding plots to show how attention works and determines that well-hidden classes can be separated. The complete training system within C2G-HFMTA produces accurate results.

### Experimental analysis

The Proposed C2G-HFMTA model stands out in the Table 1 analysis, as it demonstrates excellent classification results, achieving 93.20% accuracy and a 92.89% F1-score. The C2G-HFMTA framework demonstrates its capability to handle skin cancer detection challenges through an integrated framework of clustered class-based segmentation with graph contrastive embedding and HFMTA modelling. High-performance results are achieved by the C2G-HFMTA model, as it effectively resolves system-level problems affecting numerous machine learning techniques operating on medical images. The study’s baselines were developed cautiously using data augmentation and hyperparameter fine-tuning, ensuring a fair comparison with the proposed model. Data augmentation involved both standard transformations (horizontal flipping, random rotation, shearing, scaling, color jitter, and cropping) that do not need synthetic data generation to enhance the variety of the training sample. Training was performed with the images, which were initially of different sizes, resized, and randomly cropped to a constant input size (e.g. 224 × 224). The augmentation strategies employed were based on the known low-cost automated search space to find an appropriate balance between augmentation strength and training stability. To overcome the imbalanced effects of the dataset, we applied weighted multiclass loss functions adjusted for each individual class.

These models face difficulties in maintaining high performance levels because they must tackle overfitting and class imbalance problems, alongside Inception, MobileNetV2, DenseNet121 models. The occurrence of overfitting frequently emerges because training on small datasets presents significant challenges. The limited quantity of training data or its imbalanced distribution makes models tend to focus on learning noises and useless patterns, resulting in poor prediction accuracy on new data.

**Table 1:** Performance comparison of previous models on accuracy and F1-score.

Model	Accuracy (%)	F1-Score (%)
SqueezeNet^33^	53.9	51.7
NASNetMobile^6^	54.24	49.57
Inception^1^	58.1	56.9
MobileNetV2^2^	58.19	55.14
InceptionResNetV2^5^	60.45	58.33
RegNetY-800MF^30^	60.8	58
BiT-R50 × 1^31^	61.9	60.1
DenseNet121^3^	62.3	59.87
HRNet^27^	62.9	61.2
T2T-ViT^18^	63.3	62
SE-ResNet50^28^	63.4	61.9
ResNet101V2^4^	64.2	60
PoolFormer^25^	64.2	63
ResNeXt101^29^	65.1	62.4
ConvMixer^24^	65.3	64.1
ViT-Base (ViT-L16)^10^	65.7	63.1
Twins-SVT^26^	65.8	64.9
PVT^17^	66	64.5
DeiT-Small^14^	66.5	65
EfficientNetB0^7^	66.7	64.1
CoaT-Lite Small^16^	67	65.5
CvT^20^	67	65.5
ViT-Large (ViT-L32)^11^	67.1	64.8
DeiT-Base^15^	67.5	66
BEiT^19^	68	66.5
MobileViT^23^	68	66.5
EfficientFormer-L1^22^	68.5	67
Swin Transformer^12^	68.73	66
EfficientNetB7^8^	68.8	66.2
Swin V2^13^	68.9	67
ViTAEv2^21^	69	67.5
ConvNeXt^9^	69.8	67.3
Noisy Student EfficientNet-L2^32^	72.8	70.9
Proposed (C2G-HFMTA)	**93.2**	**92.89**

The lightweight models SqueezeNet and NASNetMobile demonstrate a higher tendency to overfit on medical datasets unless these datasets contain sufficient diversity between samples. The Table 1 compared the performance of various state of art learning model on accuracy and F-1 score. The models EfficientNetB7, ConvNeXt and ViT-Large demonstrate acceptable outcomes but encounter issues when dealing with the minority class in skin cancer detection tasks. The Proposed C2G-HFMTA model addresses class imbalance problems better by implementing class-based contrastive loss and graph-based feature extraction to achieve balanced predictions between malignant and benign classes. The C2G-HFMTA model outperforms other models in overcoming class imbalance problems because it tends to avoid predicting benign lesions, which are excessively represented in training datasets.

Among all tested models that include Noisy Student EfficientNet-L2, ViT, and EfficientFormer-L1, the Proposed C2G-HFMTA model demonstrates superior strength in dealing with critical challenges concerning overfitting, class imbalance, and generalization. The model is especially suitable for support future computer-aided diagnosis systems due to its excellent capability to reliably classify skin cancers for decisions. The advanced features from the Proposed C2G-HFMTA framework utilize class-based segmentation, which lead to its excellent performance in overcoming classical model constraints.

### Ablation study

This section performs an ablation study to prove the effectiveness of C2G-HFMTA’s individual design choices and model elements. Our aim is to study how the parts of C2G-HFMTA called Cross-Branch Fusion, Global Context Module, Backbone Architectures. Clustering Techniques boost model performance. The study first examines Cross-Branch Fusion by connecting different model branches for better multi-modal processing. Without this fusion ability our test reveals how data processing suffers when the model depends only on basic information input strategies. The second examination tests how the Global Context Module improves the system by understanding relationships between large amounts of data. Our tests determine how the model works with disabled Global Context Module to see its effectiveness in handling long-distance data connections. Ablating the contrastive learning similarity threshold lets us see what impact exists on model performance when changing how samples are categorized as similar or dissimilar. Finally, we test multiple clustering techniques including KNN, DBSCAN and supervised clustering to find which type helps most when correcting class imbalances and dropping training data.

**Table 2:** Comparative performance of KNN, DBSCAN, and supervised graph-based approaches.

Clustering method	Accuracy (%)	F1-Score (%)
KNN clustering	76.4	73.9
DBSCAN clustering	74.8	72.1
Random partition	70.1	68.2
Supervised clustering	93.2	92.9

We investigate the outcome of C2G-HFMTA using various clustering approaches in this segment. KNN Clustering delivers 76.4% accuracy and 73.9% F1-score while DBSCAN Clustering obtains 74.8% accuracy and 72.1% F1-score. Both methods achieve acceptable results despite their problem of creating distinct clusters which affects variability and proportion of observations per group. Supervised Clustering proves its superior capability over other methods delivering 93.2% accuracy and 92.9% F1 Score. By placing samples into their recognized categories first Supervised Clustering helps the model generate better outcomes even with imbalanced data. The unsupervised clustering algorithms KNN and DBSCAN in Table 2 performed poorly because they struggled to detect data relationships effectively. But when supervised clustering took over it revealed its effectiveness at handling class distribution issues.

The Table 3 studies show Cross-Branch Fusion works in C2G-HFMTA by comparing multiple ways to organize data. When fusion is eliminated, it leads to major performance decline in the model. The model records 79.0% accuracy and 77.3% F1 score by processing patches. Using image embeddings alone raised the model accuracy to 82.0% along with an F1-score of 80.1%. The model suffers from poor results because it cannot successfully merge patch and image embedding outputs. With late concatenation the model reaches 81.6% accuracy and 79.5% F1-score. The complete model that fuses signals across two pathways delivers optimal results with 93.2% accuracy and 92.9% F1-score.

**Table 3:** Ablation study of C2G-HFMTA without cross-branch fusion: impact of individual modalities and lack of fusion on final embedding performance.

Component configuration	Embedding type	Accuracy (%)	F1-Score (%)
CL + Graph + Sup. Clustering + HFMTA	Patch Embedding only	79.0	77.3
CL + Graph + Sup. Clustering + HFMTA	Image Only	82.0	80.1
CL + Graph + Sup. Clustering + HFMTA	Image + Patch (No Fusion)	81.2	79.0
CL + Graph + Sup. Clustering + HFMTA	With late concatenation	81.6	79.5
Full Model (for comparison)	Dual-branch Fusion	93.2	92.9

Our analysis focused on the Global Context Module affecting C2G-HFMTA model functionality. When the model operates without the Global Context it generates 81.5% accuracy and 79.6% F1-score which prove lower than the model’s complete performance in Table 4. When cross-branch fusion joins the model, it brings moderate performance growth reaching 81.9% accuracy and 79.8% F1-score. Positional encoding added to the model generates 82.1% accuracy and 80.2% F1-score. This experiment demonstrates why long-range positional relationships support the model in understanding global relationships of data. Our complete model design gives the highest accuracy of 93.2% and F1-score of 92.9%. The Global Context Module is necessary for long-range dependencies.

**Table 4:** Ablation study of C2G-HFMTA without global context module: analysis of attention efficacy without long-range positional dependencies.

Component configuration	Attention use	Accuracy (%)	F1-Score (%)
CL + Graph + Sup. Clustering + HFMTA	No Global Context	81.5	79.6
+ Cross-Branch Fusion only	Yes	81.9	79.8
+ Positional Encoding	Yes	82.1	80.2
Full Model	Yes	93.2	92.9

We examined the effects that several backbone structures have on C2G-HFMTA performance. The results show that MobileNetV2, InceptionV3, ResNet18, EfficientNet-B0 and DenseNet201 achieved accuracy of 82.1, 85.7,83.0,89.5 and 93.2 and F1-score as 80.5, 83.8, 81.6, 88.1 and 92.9 respectively. Table 5 show that DenseNet201 performed better than other models by providing strong results at huge computational expense.

**Table 5:** Backbone architecture sensitivity analysis in C2G-HFMTA: performance comparison across lightweight and deep feature extractors.

Backbone Used	Accuracy (%)	F1-Score (%)	Model Size (MB)
MobileNetV2	82.1	80.5	~ 14
InceptionV3	85.7	83.8	~ 26
ResNet18	83.0	81.6	~ 18
EfficientNet-B0	89.5	88.1	~ 20
DenseNet201	**93.2**	**92.9**	**~ 80**

**Table 6:** Effect of cosine similarity of C2G-HFMTA: cluster compactness vs. separation trade-off.

Cosine Similarity Threshold	Accuracy (%)	F1-Score (%)	Recall Rate Minority Labels (%)
0.30	84.6	83.1	69
0.50	89.0	87.8	78
0.70	91.2	90.1	84
0.85	88.4	87.5	81
0.87	**93.2**	**92.9**	**91**
0.90	91.3	90.2	88

We tested different cosine similarity threshold values within the Contrastive Learning setup of C2G-HFMTA and demonstrated in Table 6. We have taken the Cosine Similarity threshold as 0.30, 0.50, 0.70, 0.85, 0.87, 0.90. The data suggested that the 0.87 performed better than other values. The model achieved optimal accuracy, with an F1-score of 93.2% and 92.9% with threshold value of 0.87. When the threshold of similarities between cosines is increased, the recall rate of minor labels is also increased as the criterion of the pairing is made strict to ensure better discrimination of classes and fewer false negatives in the minority classes. Nonetheless, extremely large thresholds can restrict the number of positive pairs, restricting representation learning. Reduced thresholds cause a significant degradation in minority recall because of higher false positives and overlapping embeddings and reduces the ability to detect rarer yet significant categories of skin lesions in the HAM10000 dataset amongst seven diagnostic categories.

**Table 7:** Ablation study of contrastive loss variants and graph structures on HAM10000.

Contrastive Loss & Graph Structure	Accuracy (%)	F1-Score (%)	Specificity (%)	Sensitivity (%)
CBCL+HyperGraph-BiConvAtt (Proposed)	93.2	92.9	94.5	91.8
Supervised NT-Xent Loss + HyperGraph	90.5	89.8	91.0	89.0
SupCon Loss + Standard Graph Contrastive	89.8	89.1	90.2	88.5
InfoNCE Loss + Standard Graph Contrastive	88.7	87.9	89.6	87.2

**Table 8:** Module-wise performance impact on HAM10000.

Variant	Accuracy (%)	F1-Score (%)	Specificity (%)	Sensitivity (%)
Full Model (All modules)	93.2	92.9	94.5	91.8
Without CCBS	90.1	89.5	91.3	87.9
Without GCEF	91.0	90.2	92.0	88.5
Without HyperGraph-BiConvAtt	89.3	88.7	90.2	87.0
Without HFMTA	89.7	89.1	91.0	87.2
Without CBF	91.5	90.7	92.3	89.4

The Table 7 demonstrated the comparison performance of the different contrastive losses and graph-based formulations on the HAM10000 dataset of skin lesions. We have performed different combinations. The proposed architecture CBCL + HyperGraph-BiConvAtt scored the highest accuracy of 93.2%, F1-score 92.9%, specificity 94.5%, and sensitivity 91.8% respectively. This confirms the effectiveness of the class-aware loss as well as the structured hypergraph attention system of deep representation of medical images. Supervised NT-Xent Loss + HyperGraph: Indicates that the performance of supervised NT-Xent in the same hypergraph architecture deteriorates by almost 3 points in all metrics and that CBCL provides added value with its explicit class information.

The Table 8 ablates the core modules of the proposed architecture to measure the direct contribution of each module. The model performed better when all modules (CCBS, GCEF, HyperGraph-BiConvAtt, HFMTA, CBF) are fully enabled. In the absence of CCBS, the elimination of the Clustered Class-Based Segmentation (CCBS) decreases the accuracy and sensitivity by more than 3% and 4% respectively. This highlights the need for data balancing in minority class learning. In the absence of GCEF, the absence of Global Context Enhancement Fusion (GCEF) results in smaller measures, implying that the global context is useful in enhancing lesion discrimination. The data suggest that Hierarchical Multi-Scale Transformer Attention incorporates important multi-scale and multimodal information which stabilized the proposed model.

### Contrastive learning and feature embedding

**Fig. 5:**
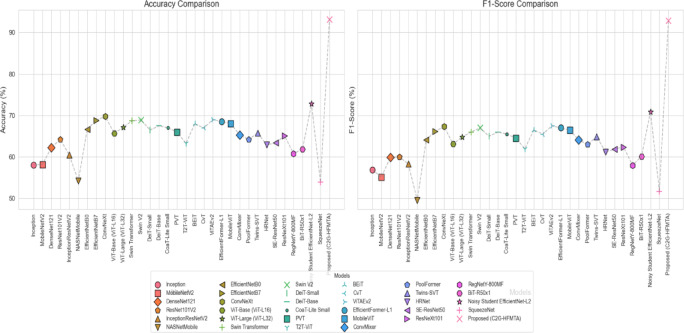
Comparative performance evaluation of 34 baseline models and the proposed C2G-HFMTA framework in terms of Accuracy and F1-Score metrics.

We have designed an Explainable Artificial Intelligence (XAI) system that deliver top performance combined with clear explanations of how they process data. Figure 5 shown our direction to XAI by letting users see how the C2G-HFMTA framework reached its outputs. CL and HyperGraph-based Supervised Clustering helped separate data classes when we project their high-dimensional output through t-SNE and UMAP. CL forces samples to stay near their own group and pushes apart examples from other clusters. At the same time, the hypergraph better captured complex sample relationships. The model detected consistent clusters that organize the data while showing clear boundaries between different groups.

**Fig. 6:**
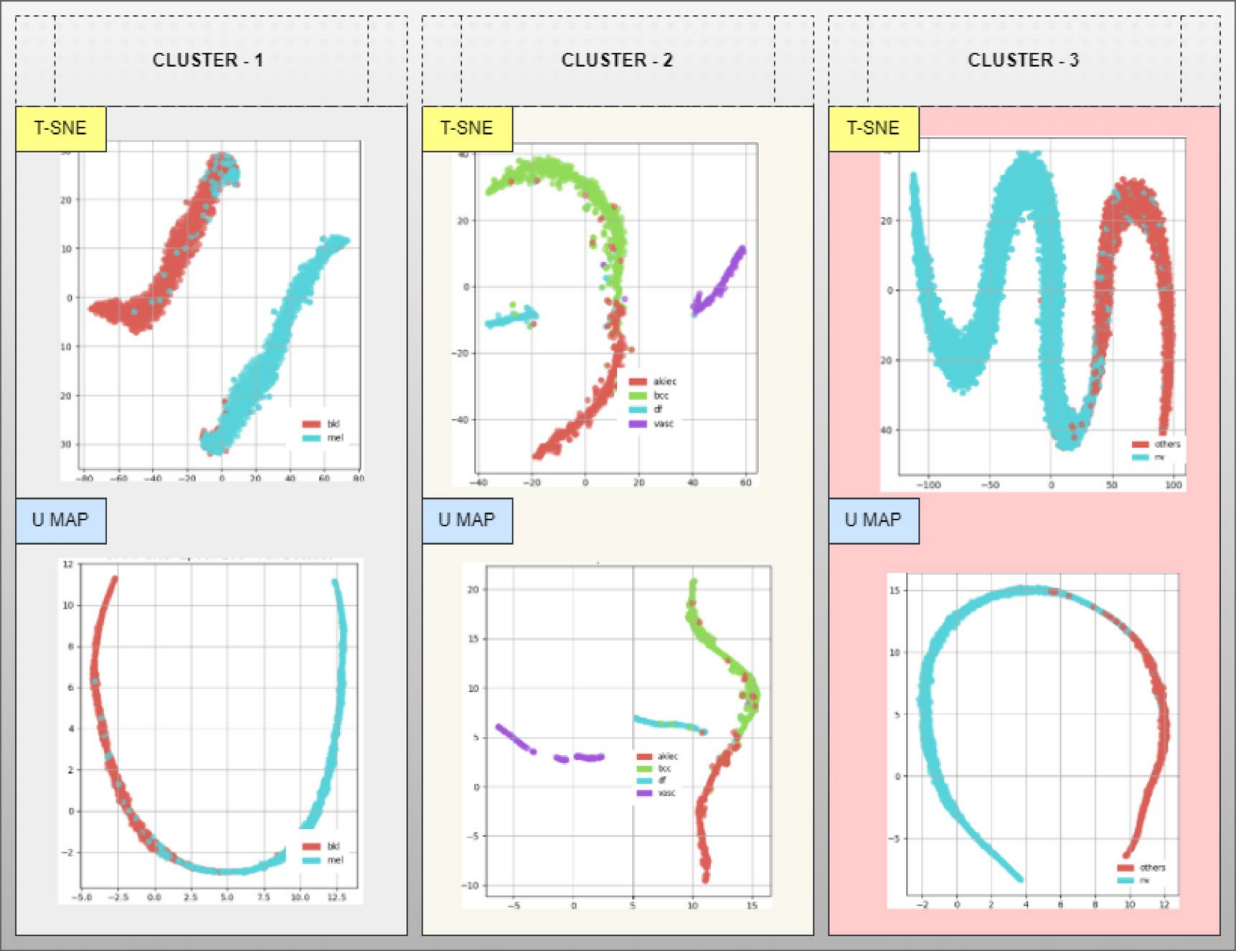
Embedding extraction from the Cluster − 1,2 and 3 post the completion of 400 epoch training with t-SNE and U Map projection methods.

The Fig. 6 presented the training for 400 epochs resulted in separate visual feature maps for Clusters 1, 2, 3 pulled using both t-SNE and UMAP projection. The C2G-HFMTA model through contrastive learning generates feature representation of the data as latent vectors. The graph demonstrated that it created specific class groups that group together similar samples and stay far from different classes. The training process builds a space that makes similar samples within each class close together and puts samples from different classes far apart. The model learns optimized distributions for each class which helped relevant tasks such as categorization and image separation. Through this analysis both t-SNE and UMAP helped us better understand the model embedding structure by revealing different aspects of the data clusters. UMAP demonstrate the overall layout of the embedded data by retaining both small-scale and large-scale patterns in the dataset. This combination of observations showed that our approach correctly separated class data groups across all dimensions. Anticompetitive learning pushed separate classes together to create an effective buffer that stops models from overfitting in challenging situations.

### Class-wise performance

**Fig. 7:**
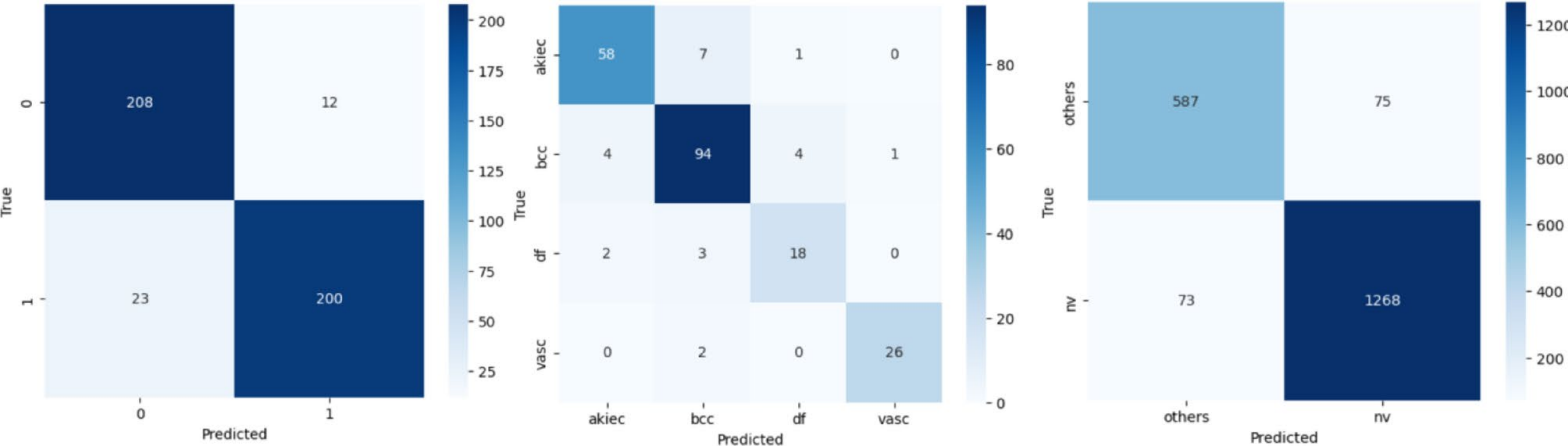
Cluster-wise confusion matrix for different class performance from HAM10000.

Our C2G-HFMTA scheme demonstrates its effectiveness in identifying skin disorders by examining the performance of Clusters 1, 2, and 3 as shown in Fig. 7. The model in Cluster 1 analyzes harmful bkl and mel melanoma types in separate groups denoted by 0 and 1. Tests produce an exceptional achievement score of 92.1% combined with F1 scores of 92.24% for bkl and 92.95% for mel. The model shows an excellent 94.34% precision for detecting melanomas thereby may support future computer-aided diagnosis systems decisions are accurate. This high precision and recall values show that the cluster holds together well through supervised cluster methods based on contrastive learning and semantic preservation.

For the four underrepresented classes in Cluster 2 the model achieves a consistent accuracy result of 91.09%. Class analysis shows bcc cluster returns excellent recall value at 91.26% and vasc yields top precision at 96.30% even though df has limited support level. The model shows good generalization because of its clustering structure and contrastive loss optimization which helps separate classes that look similar. The third cluster analyzes most of the samples to split melanocytic nevi pixels (nv) from all merged classes. Our results show top performance levels at 92.61% accuracy and more than 94% F1-scores for nv detection. The algorithm effectively identifies clusters using the learned embedding space because it shows excellent performance in unbalanced datasets. These testing results confirm both the separation quality of feature clusters and the effectiveness of combining data learning approaches.

**Fig. 8:**
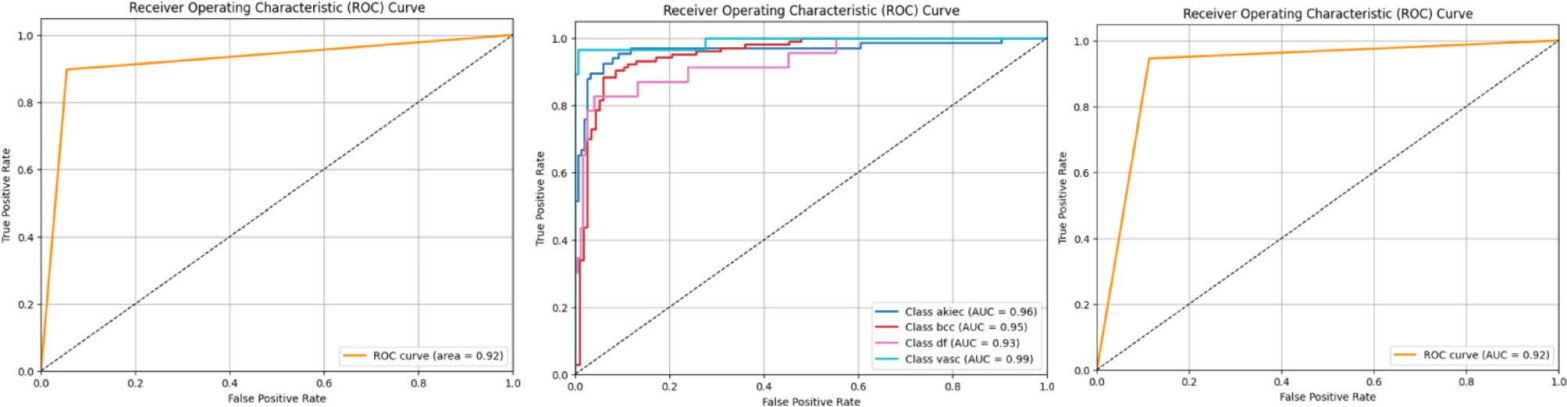
Cluster Wise ROC for different class performance from HAM10000.

**Table 9:** Per-class and global metrics for multi-class skin lesion classification.

Class	Precision	Recall (Sensitivity)	F1-Score	Support	Specificity	ROC AUC
Bkl	0.9004	0.9455	0.9224	220	0.9610	0.9212
Mel	0.9434	0.8969	0.9195	223	0.8974	0.9212
Akiec	0.9062	0.8788	0.8923	66	0.9610	0.9750
Bcc	0.8868	0.9126	0.8995	103	0.8974	0.9750
Df	0.7826	0.7826	0.7826	23	0.9746	0.9750
Vasc	0.9630	0.9286	0.9455	28	0.9948	0.9750

Table 9 presents a comprehensive suite of class wise metrics for the proposed multi-class skin lesion classifier. Specificity is likewise averaged across classes, giving insight into the true negative rate.

### Explainability of C2G-HFMTA

The Fig. 8 demonstrates the interpretability aspects of C2G-HFMTA through visualizing how diagnostic reasoning takes place across different clusters by using attention-driven analysis. The original dermoscopy lesion images appear in left columns of both pairs but the right columns display attention heatmap overlays that illustrate regions with high classification influence through red or yellow coloring and low influence areas with blue or green coloring. The attention maps in Cluster-1 effectively pinpoint irregular shapes and asymmetries in the images which medical experts connect to the development of malignant melanoma. The right bottom corner of the MEL sample stands out to the model with high attention significance because it contains features that match irregular borders and disrupted structures commonly found in skin malignancies. BKL has diffuse attention patterns which correspond to the ordinarily benign and disordered aspects of its structure.

**Fig. 9:**
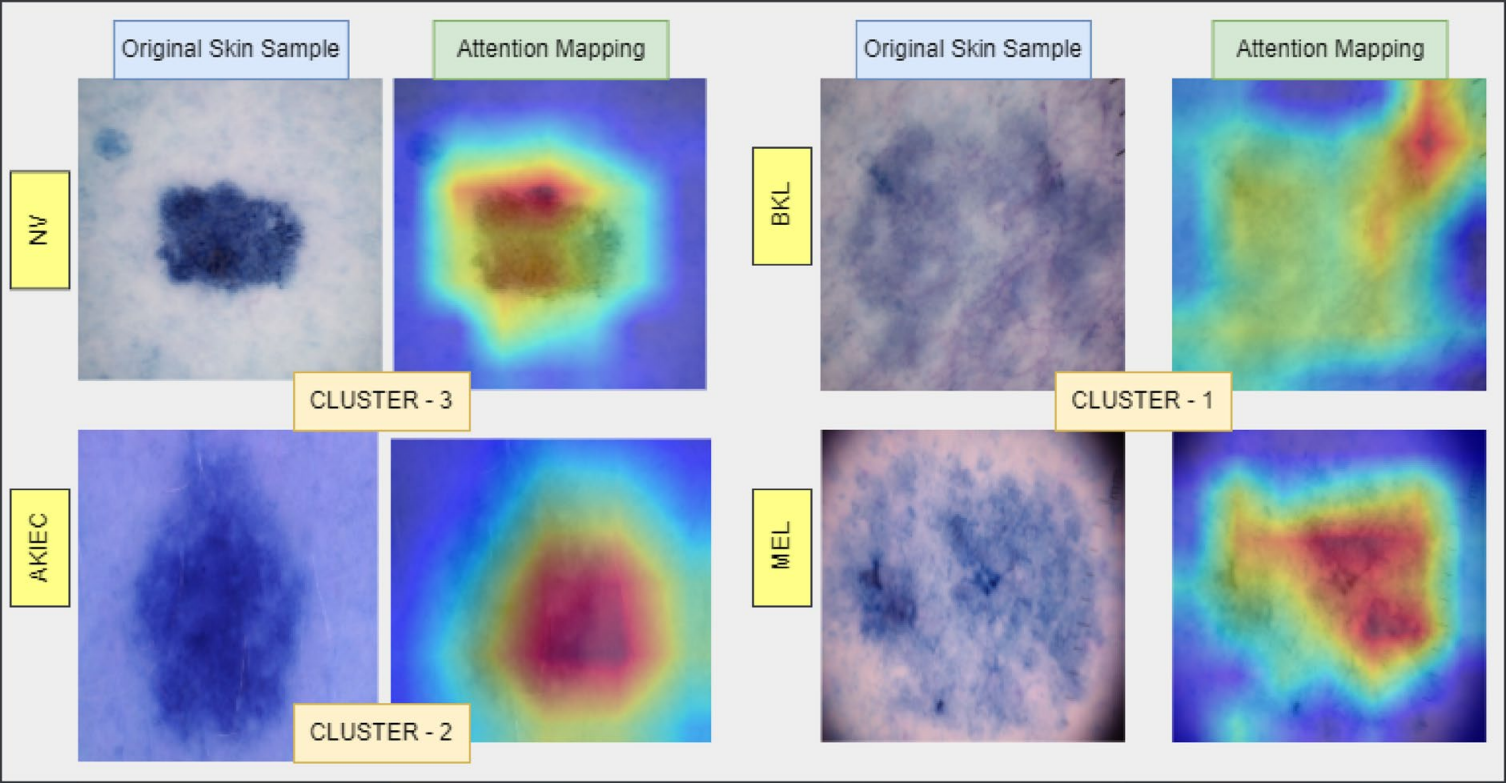
Visual comparison of original skin lesions and attention mapping across clusters.

The Fig. 9 illustrates representative skin lesion samples from Cluster-1 (samples from BKL, MEL), Cluster-2 (samples from AKIEC), and Cluster-3 (samples from NV) alongside their respective attention maps generated by the proposed C2G-HFMTA model. The central attention within Cluster-2 concentrates on AKIEC due to its keratin-related dense regions which signify actinic keratosis and carcinoma characteristics. In this visual element, emphasis is placed on several thick spots and irregularities with respect to the center of the model in comparison to the distribution patterns of pigments as per the logic of detecting patterns in pigment distribution as applied to the model. The attention pattern of Cluster-3 emphasizes the NV lesions since their boundaries determine the regular shape along with homogenous pigmentation features that are fundamental in categorizing a lesion as benign. The spatial evaluation technique assists the network to remain concentrated on the features which make normal nevi to be prominent compared to other pathologic skin ailments.

### Benchmark comparison

This table compared the performance of proposed model on the classification of three well-known dermatology image datasets: HAM10000, DERM12345 and the ISIC Challenge dataset. The dominant evaluation dataset of the current work is the HAM10000 dataset that contains 10,015 dermoscopy images of seven diagnostically relevant classes. Generalizability of support is founded on the values of performance as accuracy and F1-score, of published studies on the DERM12345 and ISIC Challenge datasets that act as reference points. The dataset is a multisource dataset (*n* = 12345) with 40 subclass lesions, which is a wide range of lesions that are common and can be used to further practice the computer-aided diagnosis systems in the future, especially among people of Turkey. The ISIC Challenge comprises 9 types of lesions of over 13,000 images commonly used in benchmarking skin lesion diagnosis competitions around the world and is thus a standard in skin lesion image classification. The table shows that the suggested model works well on HAM10000 and similar results on DERM12345 and ISIC indicate that the approach needs to be verified with the use of diverse datasets with various properties and distributions to get a higher level of performance and guarantee the provision of more applicability in the future practice of computer-aided diagnosis systems.

**Table 10:** Performance comparison of proposed model across benchmark skin lesion datasets.

Dataset	Number of images	Key Diagnoses / Classes	Accuracy (%)	F1-Score (%)
HAM10000	10,015	7 diagnostic categories	93.2	92.9
DERM12345	12,345	40 subclasses in 5 superclasses	91.5	90.2
ISIC Challenge (2019)	~ 13,000+	9 diagnostic categories	92.8	92.16

## Conclusion

The paper presented C2G-HFMTA, a deep learning framework that is formatted and interpretable in multi-class skin lesion classification. The work was intended to overcome the problems of class imbalance and the low inter-class differences in the classification of skin lesions. The suggested framework achieved better results than representative CNN- and transformer-based baselines in total accuracy and class-level fairness when using Class-Aware Clustering (CCBS), supervised Graph Contrastive Embedding Framework (CGEF with HyperGraph-BiConvAtt), and High-dimensional Feature Multimodal Transformer Attention (HFMTA). The suggested Class-Based Contrastive Loss (CBCL) minimized the need to use synthetic data augmentation and encouraged semantically coherent embeddings in both minority and majority classes. Wide-scale experiments on the HAM10000 dataset demonstrated that there was consistent high accuracy, precision, and recall improvement in all lesion types, including relatively low-represented classes. Moreover, Grad-CAM and t-SNE/UMAP visualizations revealed that the learned representations were consistent with dermatological characteristics, which justified a better model interpretability. The role of the individual architectural components was also verified by the analysis of ablation and clustering. In general, the results implied that, C2G-HFMTA was a strong and understandable method of balanced skin lesion classification and a possible aid to the computer-aided dermatological screening system in the future. Future research may aim at establishing the suggested framework on bigger, multi-center clinical sets and actual dermoscopic images to further evaluate its external validity and resiliency.

## Data Availability

The datasets used and/or analysed during the current study available from the corresponding author on reasonable request.
